# Platelet function and ageing

**DOI:** 10.1007/s00335-016-9629-8

**Published:** 2016-04-11

**Authors:** Chris I. Jones

**Affiliations:** Institute for Cardiovascular and Metabolic Research, School of Biological Sciences, University of Reading, Harborne Building, Whiteknights, Reading, Berkshire RG6 6AS UK

## Abstract

There are clear age-related changes in platelet count and function, driven by changes in hematopoietic tissue, the composition of the blood and vascular health. Platelet count remains relatively stable during middle age (25–60 years old) but falls in older people. The effect of age on platelet function is slightly less clear. The longstanding view is that platelet reactivity increases with age in an almost linear fashion. There are, however, serious limitations to the data supporting this dogma. We can conclude that platelet function increases during middle age, but little evidence exists on the changes in platelet responsiveness in old age (>75 years old). This change in platelet function is driven by differential mRNA and microRNA expression, an increase in oxidative stress and changes in platelet receptors. These age-related changes in platelets are particularly pertinent given that thrombotic disease and use of anti-platelet drugs is much more prevalent in the elderly population, yet the majority of platelet research is carried out in young to middle-aged (20–50 years old) human volunteers and young mice (2–6 months old). We know relatively little about exactly how platelets from people over 75 years old differ from those of middle-aged subjects, and we know even less about the mechanisms that drive these changes. Addressing these gaps in our knowledge will provide substantial understanding in how cell signalling changes during ageing and will enable the development of more precise anti-platelet therapies.

## Introduction

Platelets play a vital role in the chronic and acute progression of a range of diseases, most notably cardio-vascular disease (CVD). In the UK, 98 % of all patients with coronary heart disease (CHD) receive anti-platelet therapy (Nichols et al. 2012), and as a result, in 2012/2013, 38.6 million prescriptions were made for anti-platelet drugs in England alone. A figure that has increased from 3.6 million in 1991, and 18.9 million in 2001 (Bhatnagar et al. 2015). The success of current anti-platelet therapies, such as aspirin and the thienopyridine derivatives, in reducing the risk of thrombosis is supported by extensive clinical data (Antithombotic Trialists’ Collaboration 2002; Bhatt et al. 2006; Bhatt and Topol 2003; Meadows and Bhatt 2007). Thrombotic disease remains, however, a leading cause morbidity and mortality (British Heart Foundation 2011) emphasising the need for alternative or more refined therapeutic options.

One of the major challenges to improving anti-platelet therapy is balancing their anti-thrombotic potential with the risk of bleeding (Andreotti et al. 2015; Kushner et al. 2009; Serebruany et al. 2004). To get this balance right, we need to be more precise in the way we use and develop anti-platelet drugs. Patients currently receive a standard combination of anti-platelets after assessment of thrombotic risk, and/or following a thrombotic event. Individuals rarely, however, conform to a “standard”, and within a population of patients, there will be considerable variation in both their risk of thrombosis and bleeding. It is clear that better stratification of patients and the development of novel anti-platelets are needed to balance thrombotic and bleeding risk and enable better management of platelet function in selected groups. Achieving these goals requires a greater understanding of the processes that occur during platelet activation and thrombus formation, and crucially a better understanding why platelets from individuals react differently to blood vessel damage or to anti-platelet drugs.

Age is a major risk factor for CVD. In 2010, 74 % of the 180,000 people who died of CVD in the UK were over 75 years old (British Heart Foundation 2011). It is therefore in this older population that the majority of anti-platelet medication is prescribed, for whom we need to design new well-balanced, effective therapeutic strategies. Age is also a major factor giving rise to inter-individual variation in platelet count and function, and is associated with the success of anti-platelet therapy (Biino et al. 2011, 2013; Johnson et al. 1975; Meade et al. 1985b; Mohebali et al. 2014; O’Donnell et al. 2001; Segal and Moliterno 2006). This effect of ageing is particularly important because most of the work carried out to investigate platelet function, or to design and test new anti-platelet drugs, is performed in young or middle-aged healthy subjects. Yet these drugs are ultimately used in patients who are older and have enhanced risk of developing CVD.

This review focuses of the change in platelets during old age (summarised in Table 1). It will not, however, specifically address the differential effects of anti-platelet therapy in the elderly [this has been covered expertly elsewhere (Andreotti et al. 2015)], suffice to say that the age-related changes in platelet count and function detailed below are likely to have a significant impact on anti-platelet therapy as do other physiological changes that alter drug metabolism and clearance (Andreotti et al. 2015; Aymanns et al. 2010; Schmucker 2005).

**Table 1:** Summary of the age-related changes in mid- and old age

	Mid-aged Human: 30–75 years old Mouse: 10–22 months old	Old-aged Human: 75 + years old Mouse: 22 + months old
Platelet count	Stable up to about 60 years old then falls (Biino et al. 2011, 2013; Segal and Moliterno 2006; Troussard et al. 2014)	Decrease (Biino et al. 2011, 2013; Segal and Moliterno 2006; Troussard et al. 2014)
Platelet function	Increase in response (Bastyr et al. 1990; Cowman et al. 2015; Gleerup and Winther 1995; Johnson et al. 1975; Kasjanovova and Balaz 1986; Meade et al. 1985b)	???
Biochemical changes		
mRNA	Differential expression (Simon et al. 2014) Reduction in EAAT1 mRNA (Zoia et al. 2004)	???
Oxidative stress	Increase (Dayal et al. 2013)	???
PGI_2_ receptor	Decreased (Modesti et al. 1985)	???
Serotonin (5-HT)	Increase (in women no data for the change in men) (Kumar et al. 1998)	
Glutamate uptake	Decreased (Zoia et al. 2004)	???
α2-adrenergic receptor	Reduced (Supiano and Hogikyan 1993)	???

One flaw within the literature reviewed below is the lack of standardisation as to what constitutes ‘old’ in humans and mice. Definitions of what is considered old for a human have changed as society has aged, and there is no uniform definition across the literature as to what constitutes an old mouse. As a result, and as will hopefully become apparent in the course of this review, we know relatively little about how platelets from people over 75 years old differ from those of middle-aged subjects, and we know even less about the mechanisms that drive these changes.

### Platelet function

Platelets are small anucleate cells packed with complex signalling machinery that enables them to react rapidly and specifically to a variety of stimuli, most notably at sites of tissue injury. At sites of vascular damage, platelets adhere to the sub-endothelial matrix via interactions between von Willebrand Factor (VWF) and the platelet receptor complex GPIb-V-IX (Alevriadou et al. 1993; Ruggeri 2003; Wu et al. 2000). The first adherent platelets are stabilised and activated by the binding of GPVI and integrin α_2_β_1_ to exposed collagens (Siljander et al. 2004). Following this initial deposition, subsequent platelets are recruited to the forming thrombus via integrin α_IIb_β_3_ anchored tethers (Nesbitt et al. 2009). Once tethered, platelets encounter a host of agonists which are either generated and secreted by activated platelets (e.g. thromboxane A_2_) (Siess et al. 1983a, b) or released upon platelet degranulation (e.g. ADP) (Gachet et al. 1997) or synthesised on the platelet surface or at the site of thrombus formation (e.g. thrombin) (Bevers et al. 1982; Coughlin 2000). Intracellular signalling cascades initiated upon platelet activation lead to calcium mobilisation from both internal stores and the extracellular space into the cytoplasm, platelet shape change, degranulation and a change in the affinity of integrin α_IIb_β_3_ for vWF and fibrinogen (Coppinger et al. 2004; Hartwig 2006; Italiano et al. 2008; Ma et al. 2007; Varga-Szabo et al. 2009). The binding of fibrinogen to integrin α_IIb_β_3_ on different platelets supports aggregation and thrombus formation (Bennett 2001; Pytela et al. 1986), and further stimulates platelets by the activation of integrins (Clark et al. 1994; Law et al. 1999; Shattil and Newman 2004). Unfettered or inappropriate platelet activation in kept in check by a series of inhibitory signalling pathways, the most powerful of which are nitric oxide (NO) and prostacyclin (PGI2) released into the blood the healthy arterial endothelium (Jones et al. 2012).

This coordinated response enables platelets to respond rapidly to vascular damage and, in most circumstances, allows for the formation of stable thrombi that prevent excess bleeding without blocking the flow of blood past the site of damage. Changes in platelet count or responsiveness alter the dynamics of this highly regulated response, with the consequent increase in the risk of bleeding or vessel occlusion and thrombotic disease. One of the leading causes of change in platelet physiology is an individual’s age. This review will focus on the changes in platelets associated with old age because of their particular relevance to the development of thrombotic disease (the majority of which occurs in people over 75 years of age) and its treatment. The reader should also be aware that there are clear differences that exist between neonatal, infant, child and adult platelets, and these developmental changes are beyond the scope of this review and have been covered in some detail elsewhere (Bassareo et al. 2012; Ferrer-Marin et al. 2013; Haley et al. 2014; Israels and Michelson 2006).

### Change in platelet count during ageing

Platelet count shows a well-documented change during ageing. Studies of populations in Italy, France and in multiple ethnic groups within the USA show that platelet count decreases with age (Biino et al. 2011, 2013; Segal and Moliterno 2006; Troussard et al. 2014). Critically, these studies show that the effect of age is not linear. Platelet count remains relatively stable during middle age (25–60 years old) but falls in old age (60+), decreasing by approximately 8 %, or 20,000 platelets/μl, between 50- and 59-year-old subjects and those over 70 years old (Segal and Moliterno 2006). Studies in mice confirm that the change in platelet count with age is not restricted to humans. Platelet count increases in 18-month-old mice compared to mice aged 4 months old (Culmer et al. 2013; Dayal et al. 2013). However, due to the restricted age range used in most studies, a fall in platelet count in old mice has not been reported. The typical lifespan of C57BL/6J mice is 29 months, yet most studies consider 18-month-old mice to be “old-mice”, whereas they are perhaps more likely to equate to late middle-age humans.

### Change in platelet function during ageing

The effect of age on platelet function is slightly less clear. The longstanding view is that platelet reactivity increases with age in an almost linear fashion. Johnson et al. (1975) showed that platelet aggregation in response to ADP, adrenalin, collagen and arachidonic acid increased in both men and women in their early 40s compared to those in their early 20s. This finding was confirmed in 958 subjects of the Northwick Park Heart study which showed increasing aggregation in response to ADP in men and women aged 25–65 (Meade et al. 1985b), and by the work of Kasjanovova and Balaz (1986) who showed an increase in ADP and collagen induced aggregation in subject over 59 years old. Furthermore, Gleerup and Winther (1995) compared two groups of 12 healthy male subjects with a mean age of 25 and 58 years in whom the concentration of ADP needed to generate irreversible aggregation decreased with age, and Bastyr et al. (1990) showed a positive correlation between ADP-induced aggregation and age in 40 subjects aged from 22 to 62. More recently, a study of 109 healthy individuals aged 18–82 (although only 3 were older than 65) showed that platelet translocation and instability when binding to VWF in an in vitro perfusion chamber reduced with age (i.e. the ability of platelets to become activated and form stable adhesions increased with age), although total platelet surface coverage remained unchanged (Cowman et al. 2015).

These data clearly demonstrate a progressive increase in platelet responsiveness to multiple agonist during middle age (25–65 years of age) in both men and women. This data is limited, however, in what it tells us beyond this rather narrow conclusion and suffers from a number of major draw backs: (1) platelet function is usually measured by aggregation which has been shown to be confounded by a concomitant age-related increase in plasma fibrinogen levels (Meade et al. 1985a), (2) the design of these studies is such that they either have a restricted age range (i.e. they only assess changes during middle age—20 to 65 years old) (Bastyr et al. 1990; Cowman et al. 2015; Jorgensen et al. 1980; Kasjanovova and Balaz 1986; Meade et al. 1985b) or, (3) compare disparate age groups without investigating the intervening period (thereby showing that platelet function is generally higher in older subjects but not showing the time course of these changes or the change in platelet function over time in older subjects) (Gleerup and Winther 1995; Johnson et al. 1975; Reilly and FitzGerald 1986).

In light of the limitations of these studies, we must be wary of blindly supporting the dogma that has developed in the years since their publication, namely that platelet function increases with age.

Very little evidence exists on the changes in platelet function in old age (i.e. greater than 75 years old). This is partly because of an assumption that the data from middle-age subjects can be extrapolated into older age groups, but mainly because of the difficulty of studying platelet function in older subjects in whom the onset of chronic disease and the increased prescription of a range of medications make it hard to separate the effects of ageing on platelet function from other confounders. It is worth noting that while anti-platelet drugs will clearly affect platelet function, and many other common treatments, such as statins, nitrates, β-blockers or calcium channel blockers, also have a significant impact on platelet function.

Despite these difficulties, a few studies do indicate that something different may be happening during old age compared to middle age. An analysis of platelet aggregation in response to epinephrine, ADP and collagen in 1058 men and 1363 women, aged 26–82 years, from the Framingham Heart Study, concluded that “aggregability decreases with increasing age” (O’Donnell et al. 2001). In the analysis of this data, age (amongst other factors although not fibrinogen) was included as a covariate in the adjusted model. Interestingly, age was not included as a linear variable but linear spline with knots at 47 and 62 years, indicating that the effect of age on platelet function is not linear but, like the effect of age on platelet count, changes at different stages of ageing (O’Donnell et al. 2001).

A further piece of clinical evidence comes from Gilstad et al. (2009) who, unlike most studies, focused on diseased rather than healthy individuals. This has the advantage of embracing one of the potential confounders of ageing studies (i.e. the onset of disease) and enabled the recruitment of older subjects. In 54 patients aged 45–92 years with stable angina, age was negatively correlated with platelet aggregation, integrin αIIbβ3 activation and P-selectin exposure (Gilstad et al. 2009).

While these two pieces of evidence do not provide a definitive picture of the changes in platelet function occurring in old age (>75 years old), they do provide a tantalising glimpse that there may be differences between the effects of ageing on platelets in middle age compared to old age. At the very least, they suggest that basing our understanding of platelet function in the growing elderly population on an extrapolation of what occurs in middle age subjects is flawed and that there is a need for studies that specifically address this gap in our understanding.

Studies in mice, again, confirm that the change in platelet function with age is not restricted to humans. Platelet function and venous thrombus formation has been shown to increase with increasing age in mice, while time to occlusion in arterial models of thrombosis decreased (Dayal et al. 2013; Jayachandran et al. 2005). Stampfli et al. (2010) could not, however, detect a change in the occlusion time, in a photochemical induce injury model of arterial thrombosis, between 11- and 104-week-old mice (Stampfli et al. 2010). Again most studies either do not assess mice older than 18 months old, which likely equates to late middle age rather than old age, or they compare disparate age groups without investigating the intervening period. So as with humans little can be concluded with respect to old age, but we can confirm that, as with humans, platelet function in mice increases with age during middle age.

### Biochemical changes in platelets with age

Leaving aside the gaps in our knowledge of platelet function in older age, it is clear that platelets undergo significant functional changes during ageing. A number of mechanisms affecting multiple aspects of platelet signalling have been described that account for these changes.

The most obvious demonstration of how wide spread the effect of ageing is, can be seen in the age relate change in platelet mRNAs and microRNAs (Simon et al. 2014). Even though the age range over which changes were studied was relatively narrow (18–46 years old), Simon et al. (2014) still identified 129 mRNA and 15 microRNAs that were differentially expressed with age. The Gene Ontology (GO) analysis of these mRNAs and microRNAs revealed that they mapped to an array of well-established platelet function pathways, including cytoskeletal organisation, vesicle transport, signal transducer activity, ubiquitin-protein ligase activity, protein serine/threonine kinase activity and GTPase activity (Simon et al. 2014). While these age-related changes in platelet mRNA and microRNAs are clear, it is, as yet, unclear how these relate to changes in the platelet proteome during ageing.

In line with many other tissue types, oxidative stress has also been shown to increase in platelets during ageing. The results of which, including modulation of the ability of NO to inhibit platelet activation, may explain some of the phenotypic variations seen in platelet during ageing. Accumulation of platelet hydrogen peroxide (H_2_O_2_) and the up-regulation of platelet P47^phox^ and Sod1 mRNA has been demonstrated in 18-month-old mice compared to 4-month-old mice, suggesting that pathways involving NADPH oxidase and SOD1 lead to the increased generation of H_2_O_2_ (Dayal et al. 2013). Furthermore, the levels of thioredoxin interacting protein (TXNIP), a negative regulator of thioredoxin-1 (TRX-1) which has wide oxidoreductase activity including the reduction of peroxides (Nordberg and Arner 2001), has also been shown to be increased in older subjects (mean age 67 years) compared to younger subjects (mean age 27 years) (Sverdlov et al. 2013). Together the increase in peroxides with age due to either increased generation or the decrease in their reduction is likely to promote platelet reactivity during ageing. Indeed Apocynin, an inhibitor of NADPH oxidase, inhibited the increase in platelet activation seen in 18-month-old mice, as did the overexpression of glutathione peroxidase-1 (Gpx1) which reduces and detoxifies peroxides (Dayal et al. 2013).

A number of other specific changes in platelets of older subjects have also been noted; increased age also correlates with a reduction in the platelet receptors for PGI_2_ (Modesti et al. 1985), and serotonin (5-hydroxytryptamine, 5-HT) as well as its metabolite 5-hydroxyindoleacetic acid (5-HIAA) was shown to be higher in the platelets of older women (Kumar et al. 1998). Interestingly, while the concentration of 5-HT in platelets was positively correlated with age in women between 40 and 84 years old, the concentration of 5-HT in the plasma of the same women was negatively correlated with age (Kumar et al. 1998).

In opposition to the general trend of increased platelet activity in older subjects, there are a few notable changes that have been demonstrated to have the opposite effect. Glutamate uptake and the expression and mRNA levels of the glutamate transporter EAAT1 were reduced in platelets from old (mean age 74 years) compared with young (mean age 41 years) human subjects (Zoia et al. 2004). While it is more widely known for its role as a neurotransmitter, glutamate has also been shown to contribute to platelet activation. Released from platelet granules upon stimulation, glutamate binds to the alpha-amino-3-hydroxy-5-methyl-4-isoxazolepropionic acid (AMPA) receptor (AMPAR). The result of which is enhanced platelet activation and increased thrombus formation (Morrell et al. 2008).

Also the density of the high affinity α2-adrenergic receptor has been shown to be lower in platelet from old (61–75 years) subjects compared to young (19–31 years) subjects which corresponded with decreased sensitivity to epinephrine (Supiano and Hogikyan 1993).

Taken collectively, these data demonstrate that no one change is responsible for the age-related variation in platelet physiology. Rather a range of pathways, having both positive and negative effects, combine, the end result of which is a gradual increase in platelet function during middle age. As with our general understanding of platelet function in old age, there are relatively little data on the biochemical changes that occur in platelets of the elderly (over 75 years old in humans and over 22 months old in mice) which severely limits the conclusions we can draw.

### What drives these changes?

The changes seen in platelets during ageing are likely to result from alterations in platelet production and a reaction to the environmental changes within the blood or vasculature. Age-related changes in the bone marrow (the site of platelet production) have been documented for decades (Custer and Ahlfeldt 1932; Hartsock et al. 1965). In humans, the amount of hematopoietic tissue within the bone marrow and its cellularity remain relatively stable during middle age but drops in subjects over 80 years old, when there is also a marked increase in apoptotic cells within the bone marrow (Hartsock et al. 1965; Ogawa et al. 2000). Similarly in mice, while hematopoietic stem cell (HSC) numbers increase with age, there is a reduction in their functional activity and a general impairment of the hematopoietic system (Flach et al. 2014; Rossi et al. 2008). This leads in ageing to reduced numbers of leukocytes and lymphoid cells, reduced erythropoiesis, but increased numbers of myeloid cells (Florian et al. 2012). These changes may in part result from an increase in Cdc42 signalling in long-term HSCs from old mice (20–24 months old) driven by an as yet unexplained increase in their expression of Wnt5a (Florian et al. 2012, 2013).

Changes in hematopoietic tissues will undoubtedly have a major impact of platelet count, but possibly also function, and they may well explain the fall in platelet count seen during ageing. The time scales, however, over which these two changes are reported to occur in the literature do not quite match. The fall in platelet count starts in humans over 60 years old, whereas the measurable change in human hematopoietic tissue occurs later. This difference may simply be due to dissimilarities in the subjects used in these various studies or an inability to detect more subtle changes in hematopoietic tissue. There remains, however, a dearth of specific data regarding the exact changes in megakaryocytes over these time scales. It is, therefore, hard to draw firm conclusions. Certainly, there are little or no data that link the changes in platelet transcriptome (which was observed in 18- to 46-year-old subjects) and the increase in platelet function in middle age, to changes in megakaryopoiesis or platelet production.

Platelet activity and, to a certain extent, count will also be a product of the environment within which they circulate over their 10-day lifespan. It is known that plasma composition and endothelial function change with age in ways that may affect platelet function. For example, clinical chemistry analysis of plasma from 3- and 22-month-old mice showed a range of differences including an increase in free fatty acid and decrease in triglyceride levels with age (Houtkooper et al. 2011). It is, also, well documented that endothelial dysfunction increases with age, leading to reduced nitric oxide (NO) bioavailability and altered prostaglandin profiles (Qian et al. 2012; Tschudi et al. 1996; Walsh et al. 2009; Yavuz et al. 2008). As previously mentioned, NO and PGI2 are potent inhibitors of platelet function. The gradual reduction in their bioavailability may lead, in middle age, to increased platelet function. This reduction in platelet inhibition could also lead to a fall in platelet count at the point where deterioration in vascular health prompts significant number of platelets become sufficiently activated to bind to sites of impaired endothelial function or vascular damage, or be removed from the circulation in the liver or spleen.

Conversely, there are also elements of vascular ageing that may inhibit platelet function. For example, a typical feature of vascular ageing and the progression of a range of vascular diseases are the degradation of extracellular matrix and the release of elastin-derived peptides (EDP) into the circulation (Antonicelli et al. 2007; Fulop et al. 2012; Maurice et al. 2013). Recent work has shown that kappa-elastin (a complex mixture of approximately 29 EDPs) inhibited platelet aggregation to multiple agonists and prolonged occlusion time in a FeCl_3_-induced model of thrombosis in mouse mesenteric arterioles (Kawecki et al. 2014).

It seems likely that a combination of age-related changes in the bone marrow, in megakaryopoiesis, platelet production and a change in the conditions platelet experience within the circulation play a part in regulating the modification of platelets during ageing. There are, however, very little definitive data to show that these changes occur at the ages when platelet count drops or platelet function increases, or how they alter platelet production, function or lifespan.

### The impact of disease on age-related platelet changes

In the majority of the human population, ageing is also accompanied by the onset of diseases which also impact on platelet biology. For example, evidence from patients with Non-Alcoholic Fatty Liver Disease (NAFLD) suggests that this may alter or perhaps drive age-related changes in platelet count (Sung et al. 2012). In a study of 2586 people examined at baseline and after 4 years, Sung et al. showed that increased platelet count at baseline was a risk factor for developing NAFLD, but at follow-up, those subjects who had developed NAFLD had a lower platelet count (Sung et al. 2012). It has also been shown that platelet turnover is higher in diabetic mice (Hernandez Vera et al. 2012) and is also higher in stable CAD patients who are diabetic compared to these patients who are not diabetic (Larsen et al. 2014). We must therefore be sensitive when interpreting the age-related platelet changes to the chronic conditions that develop, often at sub-clinical levels, over the same time scales.

## Conclusion

There are clear age-related changes in platelet count and function (Table 1), driven by changes in hematopoietic tissue, the composition of the blood and vascular health. There is differential mRNA and microRNA expression, an increase in oxidative stress and changes in platelet receptors. These changes affect both bleeding and thrombosis and are particularly pertinent given that thrombotic disease and use of anti-platelet drugs is much more prevalent in the elderly population, yet the majority of platelet research is carried out in young to middle age (20–50 years old) human volunteers and young mice (2–6 months old). As this review has hopefully demonstrated a clear need for more clarity and detailed research in this area, one major flaw within the literature reviewed here is the lack of standardisation as to what constitutes ‘old’ in humans and mice. Definitions laid down in the 1960s and 1970s when perhaps 65 to 70 years old was considered old, no longer meets the reality of most subjects receiving anti-platelet therapy. Similarly stating that an 18-month-old mouse is ‘old’ because it is considerably older than the 4-month-old control mouse, does not mean it old in terms of getting to the end of its expected lifespan. As a result, we know relatively little about exactly how platelets from people over 75 years old differ from those of middle-aged subjects, and we know even less about the mechanisms that drive these changes.

Platelets are easily obtained from subjects or patients making them a potentially useful marker for changes in the vascular or haematopoietic environment. It seems likely that the age-related change in platelet parameters, outlined above, will vary between individuals both in terms of the magnitude of the changes and the time scale over which these changes take place. It may, therefore, be useful to know how an individual’s platelet parameters change over time rather than focusing on one off platelet measurements that are compared to a population normal range, as is currently most often the case. Knowing how an individual’s platelet count and function change over time may, potentially, be a useful marker for the early onset of age and disease-related vascular changes, and perhaps an effective way to improve the precision of anti-platelet therapy.

Understanding the changes effecting platelets during ageing, the mechanisms that drive these changes and how they are affected by disease will enable us to understand the broader physiological effects of ageing on platelets and cell signalling more generally. It is also likely to enable the development of more precise anti-platelet therapies.
